# Single-cell RNA sequencing reveals pro-tumorigenic intestinal mast cell programs in colorectal cancer

**DOI:** 10.1038/s41419-026-09183-1

**Published:** 2026-08-12

**Authors:** Erisa Putro, Alessia Carnevale, Caterina Marangio, Giuseppe Pietropaolo, Nadia Domenica Milito, Giovanna Peruzzi, Cinzia Fionda, Helena Stabile, Francesca Sozio, Angela Santoni, Giuseppe Sciumè, Valerio Fulci, Rosa Molfetta, Rossella Paolini

**Affiliations:** 1 Department of Molecular Medicine, Laboratory affiliated to Istituto Pasteur Italia - Fondazione Cenci Bolognetti, Sapienza University of Rome, Rome, Italy; 2 IRCCS Neuromed, Pozzilli, Italy; 3 Center for Life Nano & Neuro Science, Istituto Italiano di Tecnologia, Rome, Italy; 4 Present Address: Department of Molecular and Clinical Medicine, Sapienza University of Rome, Rome, Italy

**Keywords:** Tumour immunology, Colorectal cancer

## Abstract

Mast cells (MCs) are multifunctional immune cells with context-dependent functions in cancer. In colorectal cancer (CRC), their contribution remains debated, suggesting that distinct MC subsets may either support tumor progression or promote anti-tumor immunity. Using single-cell RNA sequencing in a mouse model of inflammation-driven CRC, we uncovered extensive MC plasticity during tumor progression. Transcriptomic profiling and pseudotime trajectory analysis revealed a transition from precursor-like MCs exclusively present in adjacent tissue to differentiated tumor-associated MC (TAMC) subsets. TAMCs displayed a distinct repertoire of proteases and pro-inflammatory cytokines, contributing to increased vascular permeability and recruitment of additional immune cells. Moreover, TAMCs exhibited upregulation of molecules with immunosuppressive potential and underwent a tumor microenvironment (TME)-driven metabolic reprogramming. Accordingly, antibody-mediated MC depletion reduced tumor burden. Notably, most of the transcriptional features identified in a murine CRC model were recapitulated in human CRC, supporting the translational relevance of this MC program. Our findings identify a tumor-adapted MC state that orchestrates immune evasion and tissue remodeling during CRC progression, supporting the notion that MC are reprogrammed toward an immune-suppressive and pro-tumorigenic phenotype.

## Introduction

Colorectal cancer (CRC) is the third most common malignancy and the second leading cause of cancer-related global death [1, 2]. Beyond genetic and epigenetic alterations, chronic intestinal inflammation represents a major risk factor for CRC, as in inflammatory bowel diseases, where patients frequently develop colitis-associated CRC [3]. The initiation and progression of CRC are profoundly influenced by immune cells, which orchestrate both pro- and anti-tumor responses within the tumor microenvironment (TME) [4].

Mast cells (MCs) are tissue-resident immune sentinels abundantly distributed in the intestinal mucosa and submucosa, where they play a crucial role in maintaining epithelial integrity at the host-environment interface [5]. In mice, intestinal MCs are broadly categorized into two subtypes: connective tissue-like MCs (CTMCs), which express mMCP4, mMCP5, mMCP6, and mMCP7 found in gut submucosa; mucosal MCs (MMCs), characterized by mMCP1 and mMCP2 expression, and located near the intestinal epithelium [6, 7].

Similarly, in the human gastrointestinal tract, MCs present in the lamina propria are tryptase-positive and chymase-negative, while the main MC population in the intestinal submucosa expresses both tryptase and chymase [8, 9].

This classification does not reflect the high level of intestinal MC plasticity [10]. Of note, recent advances in single-cell RNA sequencing (scRNA-seq) have uncovered distinct MC subpopulations, each with unique transcriptional profiles and functional properties [11–13]. Due to the constant change in the gut microenvironment, intestinal MCs act as dynamic modulators of homeostasis and disease progression through the release of a diverse set of mediators such as proteases, cytokines, chemokines, and lipid mediators, that regulate tissue remodeling, angiogenesis, and mucosal immunity [10, 14]. However, the specific contributions of different intestinal MC subsets during CRC development have not yet been fully characterized [11, 15–19].

In this study, we combine the azoxymethane (AOM)/dextran sodium sulfate (DSS) model [20] with single-cell RNA sequencing to dissect MC heterogeneity in inflammation-driven CRC. We identified distinct MC subsets, including a tumor-associated, mature population with unique transcriptional, metabolic, and immunomodulatory features. Using integrative analyses and functional validation, we demonstrate that TAMCs adopt a specialized functional state characterized by tissue-specific interactions and pro-inflammatory cytokine secretion. These properties are confirmed by conserved transcriptional programs in human CRC, uncovering a previously underappreciated role for TAMCs in regulating the colorectal tumor microenvironment.

## Results

### MC heterogeneity in the AOM/DSS murine model of CRC

**Fig. 1:**
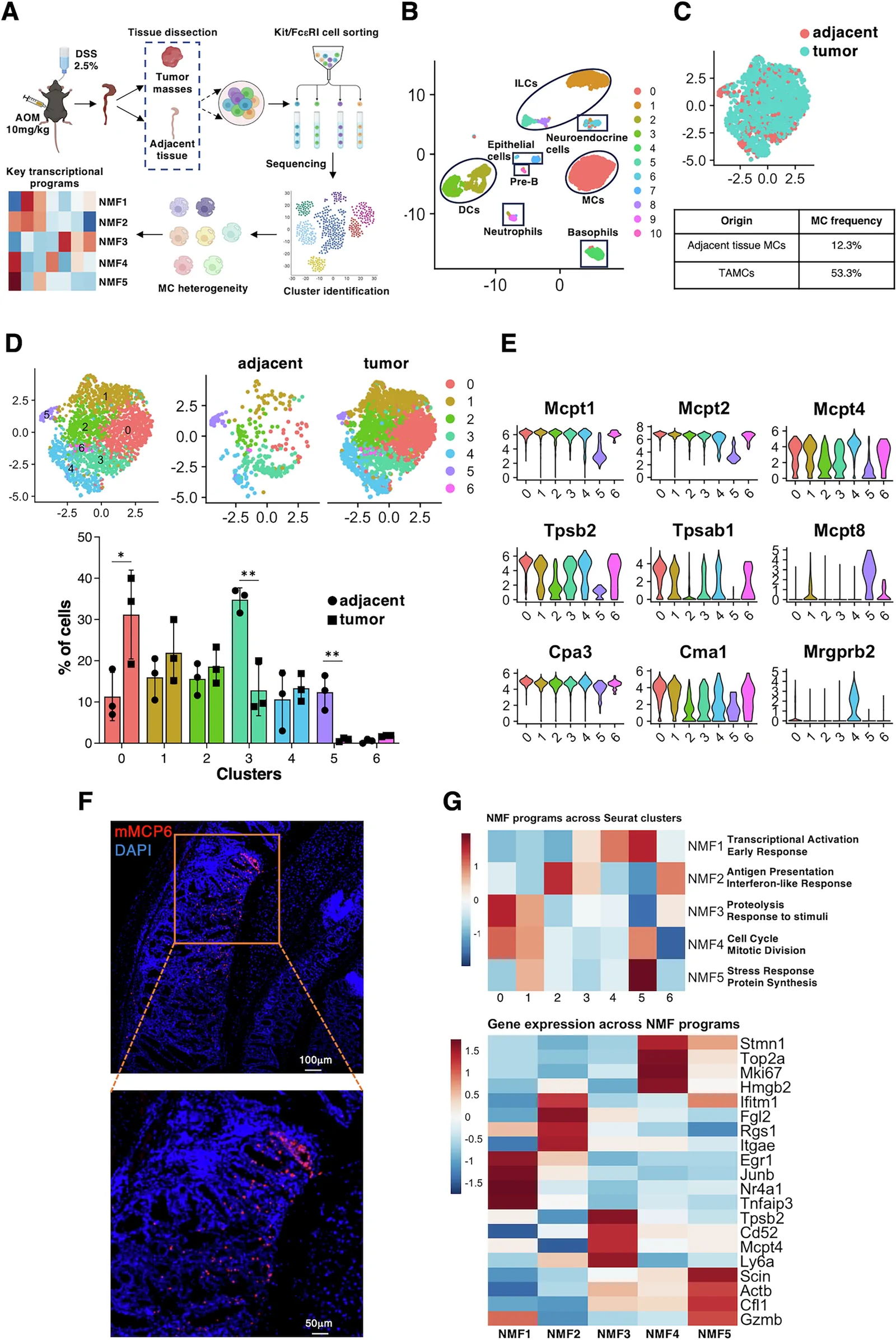
Unsupervised clustering and characterization of the mast cell (MC) landscape in AOM/DSS-treated mice. **A** Experimental and computational workflow created with BioRender.com. **B** UMAP plot based on unsupervised clustering and 0.2 resolution showing 11 clusters and 8 lineages, as indicated. Each dot represents a single cell, with color-coded according to the cell types as indicated in the legend to the right. **C** UMAP plot showing the distribution of MCs within the dataset and divided by sample type. Table summarizes the frequency of MCs within each sample type. **D** UMAP plot showing the sub-clustering of MCs. Cells are color-coded according to their cluster identities (0–6) and separated based in adjacent and tumor tissue, as indicated. Histograms report the frequency of each MC cluster present in the tumor and adjacent tumor-free tissue. (means ± SD of three samples of 6 mice/sample). Paired Student’s *t* test: **P < 0.01, *P < 0.05. **E** Violin plots illustrating the expression levels of key MC proteases and markers across the identified clusters (0–6). The genes displayed include: Mcpt1, Mcpt2, and Mcpt4, Tpsb2 and Tpsab1 (proteases MCP6 and 7, respectively), Mcpt8 (MC precursor marker), Cpa3 and Cma1 (carboxypeptidase A and chymase MCP5, respectively), Mrgprb2 (a marker for a subpopulation of CTMCs). **F** Colon paraffin-embedded sections from AOM/DSS-treated mice were stained with anti-mMCP6, followed by Alexa-fluor 594 secondary antibody (red). Cell nuclei were counterstained with DAPI (blue). Images were acquired with a Zeiss LSM980 confocal microscope using a ×20/0.8NA objective and are representative of three independent experiments. **G** Program modules of MCs in adjacent tumor-free and tumor tissue. Modules were clustered from five expression programs identified by the NMF method. Upper, the *x* axis represents MC clusters, and the *y* axis represents the programs from 1 to 5. Lower, the *x* axis represents NMF programs, and the *y* axis represents the single genes per NMF.

To investigate the MC landscape in CRC, we performed single-cell RNA sequencing from tumor and adjacent non-transformed colon tissue (Fig. 1A). To overcome the low abundance of intestinal MCs, we isolated c-Kit⁺ and FcεRIα⁺ cells, including both single- and double-positive populations (Fig. S1). A total of 6253 single cells were analyzed: 3813 cells from tumor masses, while 2440 cells from adjacent non-transformed tissue. Unsupervised analysis identified eleven cell clusters that were classified in eight lineages based on specific markers (Figs. 1B and S2A). The Uniform Manifold Approximation and Projection (UMAP) approach displayed one unique MC cluster distinguished based on the expression of canonical MC-specific proteases, including *Mcpt1*, *Cpa3*, and *Tpsb2* (Fig. S2B). MC subsetting revealed a marked increase in the TME compared with adjacent non-transformed tissue (Fig. 1C). Unsupervised clustering revealed seven MC clusters (from 0 to 6) (Fig. 1D, upper panels). Cluster 0 was enriched in TAMCs, whereas cluster 3 significantly decreased in tumor tissue, and cluster 5 was exclusively detected in adjacent non-transformed tissue (Fig. 1D, lower panel).

Mucosal protease transcripts *Mcpt1* and *Mcpt2* were uniformly expressed across MC clusters, whereas connective tissue and precursor proteases (*Mcpt4*, *Tpsb2*, *Tpsab1, Cma1*, and *Mcpt8)* showed heterogeneous expression (Fig. 1E). Cluster 0 displayed high *Tpsb2* and *Mcpt4* expression, encoding mMCP6 and mMCP4, consistent with a tumor-associated expansion of connective tissue-like MCs, as previously reported [21]. Enrichment of this population in transformed colonic tissue was supported by immunofluorescence for mMCP6 (Fig. 1F) and mMCP4 and their strong colocalization (Fig. S3A), whereas mMCP6^+^ MC accumulation was not observed in DSS-induced acute colitis (data not shown). Cluster 5, differently from all the other clusters, showed low levels of mucosal and connective tissue-like protease transcripts, but elevated *Mcpt8* levels (Fig. 1E). This protease is characteristic of bipotent MC-basophil progenitors; its expression is lost during MC differentiation while being retained in mature basophils [22]. To exclude the contamination of differentiated basophils, we verified that cluster 5 did not express specific mature basophil markers such as *Cebpa* and *Itga2/Cd49b* but showed high expression of MC-basophil precursor markers, including *Cd9*, *Gzmb*, and *Stat5b* (Fig. S3B).

These data demonstrate a previously unappreciated heterogeneity of MCs in the AOM/DSS CRC model, dominated by the population of mMCP6⁺ MCs within tumor tissue.

### MC clusters are associated with different transcriptional programs

To identify transcriptional pathways specifically upregulated for MC clusters, we applied non-negative matrix factorization (NMF) and identified 5 robust expression programs (Fig. 1G, upper panel).

Cluster 0 resulted in enrichment in NMF3 (response to external stimuli and extracellular protein degradation), containing classical MC proteases (*Tpsb2* and *Mcpt4)* and expressed *Cd52* and *Ly6a* markers (Fig. 1G, lower panel). In contrast, cluster 5 was characterized by Stress Response and Protein Synthesis (NMF5) pathways that included stress-related and ribosomal genes, cytoskeletal regulators (*Scin, Cfl1*), and *Gzmb*, suggesting altered cytotoxic potential or translational reprogramming. This cluster was also enriched in NMF1, associated with Transcriptional Activation and Early Response genes (*Junb, Egr1, Nr4a1*) and NF-κB regulators (*Tnfaip3*), consistent with an activated MC state.

NMF4, shared by cluster 0 and cluster 5, comprised cell cycle-related genes (*Mki67*, *Top2a*, and Stmn1) and chromatin and cytoskeletal regulators *(Hmgb2)*, supporting the presence of proliferative MCs in both tumor and tumor-free tissue.

Analysis by tissue distribution (tumor versus adjacent tissue) showed a strong enrichment of NMF3 in TAMCs (Fig. S4), consistent with its predominant association with cluster 0.

Therefore, we report the presence of MC clusters exhibiting unique transcriptional programs, providing compelling evidence of MC reprogramming in the TME.

### MCs enriched in tumor tissue show a mature phenotype

**Fig. 2:**
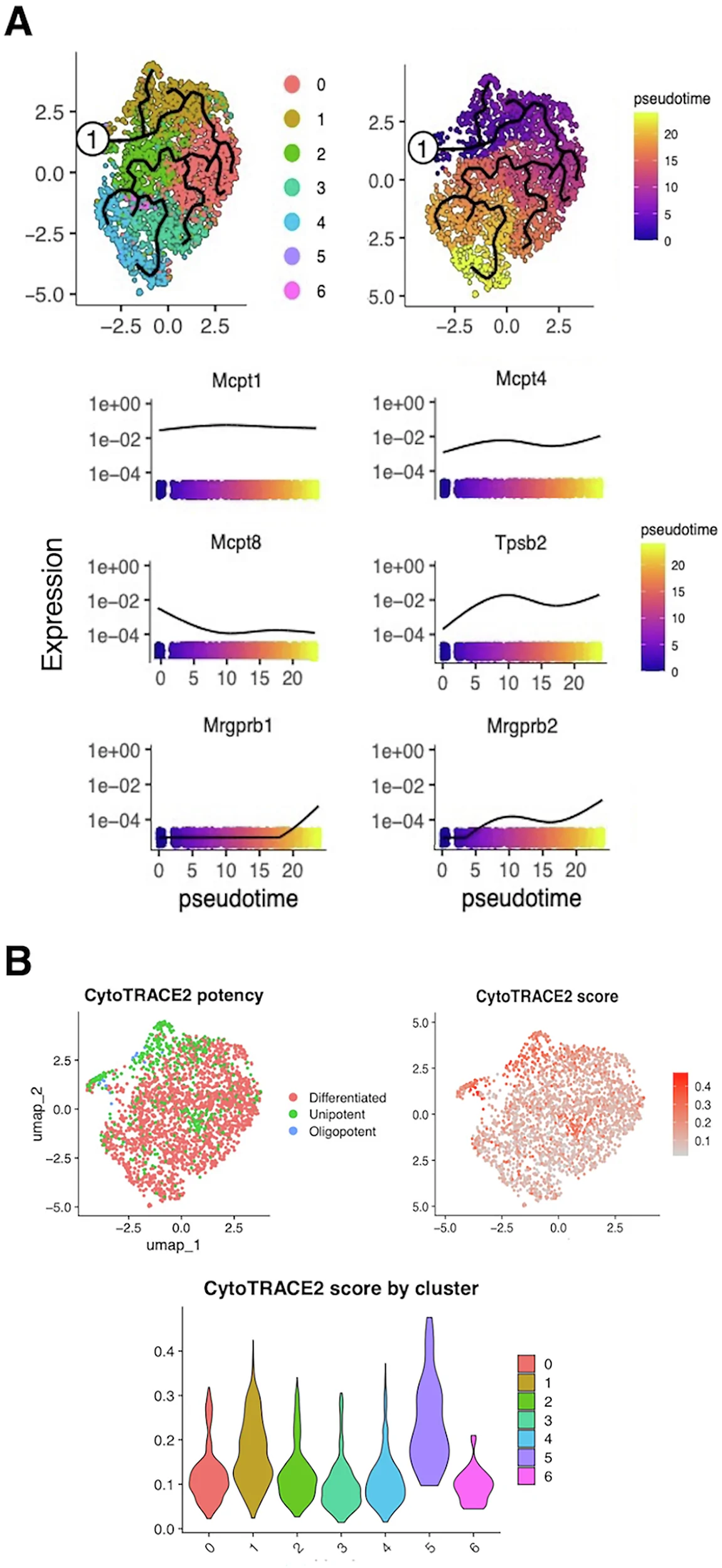
Pseudotime analysis of scRNAseq dataset. **A** Upper panels: UMAP projection of MC subclusters (left) colored by pseudo-temporal trajectory (right). Cluster 5 was chosen as the starting point (number 1). Lower panels: Kinetics of Mcpt1, Mcpt8, Mcpt4, Tpsb2, Mrgprb1 and Mrgprb2 expression levels along pseudotime trajectory. **B** CytoTRACE2 analysis of MC differentiation potential. UMAP visualization of CytoTRACE2 potency states (left) and CytoTRACE2 scores (right), together with cluster-wise score distributions (lower panel). Higher CytoTRACE2 scores indicate increased developmental potential.

To evaluate the maturation states among the different MC clusters, we performed pseudotime trajectory analysis that orders cells from immature to mature phenotype. This analysis revealed a clear maturation gradient among the different MC populations, with early pseudotime states enriched in MCp (chosen as the starting point, cluster 5) and late states associated with mature MC populations (clusters 0, 3, and 4) (Fig. 2A, upper panels). The expression of canonical MC proteases evolved along the trajectory (Fig. 2A, lower panels): *Mcpt8* was enriched in early pseudotime, while *Mcpt4* and *Tpsb2* increased toward later stages, indicating a progressive phenotypical maturation. Notably, cluster 0, highly abundant in the tumor, occupied some of the terminal branches of the trajectory, further supporting its fully differentiated identity. Cluster 4 formed a distinct terminal branch in the pseudotime trajectory and was defined by high expression of *Mrgprb1* and *Mrgprb2* transcripts, encoding for two receptors of the Mrg family previously associated with mature connective tissue-like MCs [12]. Interestingly, this cluster was equally represented in both tumor and adjacent non-transformed tissues (see Fig. 1D), suggesting that it may represent a resident, tissue-stable population of mature MCs with connective tissue-like features. To further support the MC differentiation trajectory, we performed a complementary analysis using CytoTRACE2 (Fig. 2B). Consistent with the pseudotime analysis, cluster 5, followed by cluster 1, exhibited the highest developmental potential, whereas the remaining clusters displayed progressively lower potency scores, supporting the existence of a differentiation continuum among MC subsets.

**Fig. 3:**
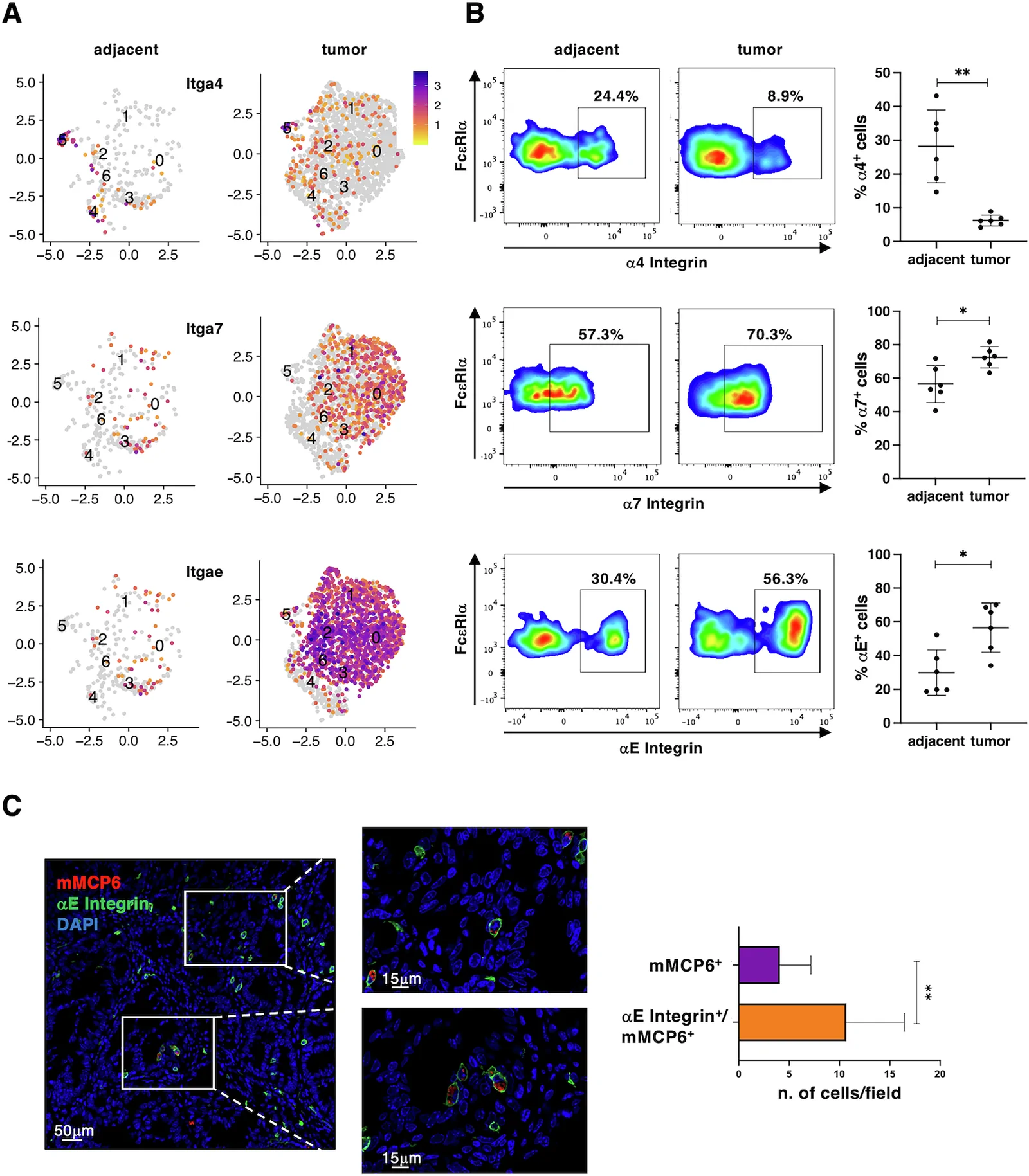
Integrin expression in TAMCs. **A** UMAP projection of integrin expression separated based on sample type (adjacent versus tumor). **B** Flow cytometry analysis of α4, α7, and αE integrin subunits on MCs isolated from adjacent non-transformed and tumor tissue from 6 mice. Representative plots on the left and statistical analysis on the right of each panel. Paired Student’s *t* test: **P < 0.01 and *P < 0.05. **C** Colon paraffin-embedded sections from tumor tissue of AOM/DSS-treated mice were double-stained with anti-mMCP6, followed by AlexaFluor 594 (red) and anti-αE integrin antibody, followed by AlexaFluor 488 secondary antibody (green). Cell nuclei were counterstained with DAPI (blue). Images were acquired with a Zeiss LSM980 confocal microscope using a ×20/0.8NA objective or a ×63/1.3NA objective (inserts). Quantification of mMCP6⁺ MCs and double-positive mMCP6⁺/αE integrin⁺ cells was performed using Fiji ImageJ in 10 randomly selected fields from three independent experiments. Paired Student’s *t* test: **P < 0.01.

We also examined the expression of integrins previously associated with MC maturation [23–25] (Fig. 3A). *Itga4* transcript, encoding the α4 integrin which facilitates the homing of MCps to the intestine [23], was predominantly expressed in cells belonging to cluster 5. In contrast, α7 integrin (*Itga7*), binding extracellular matrix laminins, and αE integrin (*Itgae*), required for MC maturation and retention within the intestinal epithelium [24, 25], was upregulated in multiple clusters, likely representing common features of maturation and epithelial localization. Accordingly, flow cytometry analysis revealed a decreased expression level of the integrin α4 and a concomitant increase of the integrins α7 and αE in TAMCs compared to adjacent tissue (Fig. 3B). Notably, confocal microscopy showed a high proportion of mMCP6⁺ MCs co-expressed αE integrin within tumor regions (Fig. 3C). Overall, our results demonstrate a progression towards a mature MC phenotype within tumor tissues, characterized by the accumulation of connective tissue-like MCs in the TME.

### Tumor-associated MCs establish strong interactions with the stromal compartment

**Fig. 4:**
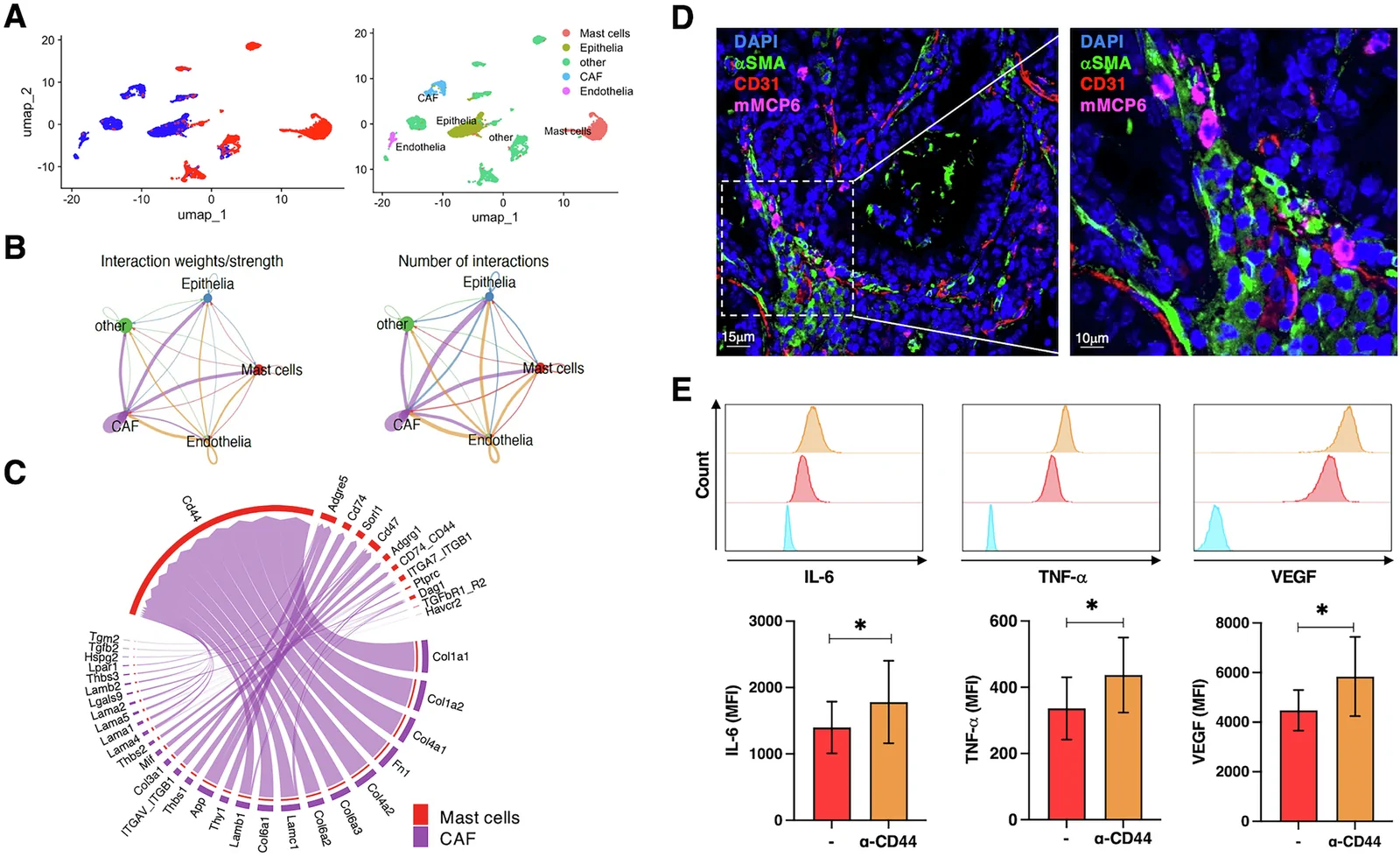
Analysis of MC communication with epithelial and stromal cells. **A** UMAP visualization of the tumor integrated dataset. The left panel shows cells derived from tumor tissue in our dataset represented in red and cells from a publicly available AOM/DSS colorectal cancer dataset (GSE134255) represented in blue. The right panel shows the identification of 9 populations in the integrated datasets, including epithelial subsets, MCs, fibroblasts, and endothelial cells. **B** Circle plots showing the number and strength of interactions stratified in tumor tissue populations. The thickness of the line indicates a greater number/communication probability. **C** CellChat cell–cell communication networks inferred from expression of ligand-receptor pairs. Chord diagrams showing communication between MCs and CAFs. **D** Colon paraffin-embedded sections from tumor tissue of AOM/DSS-treated mice were stained with anti-CD31 followed by AlexaFluor 647 secondary antibody (red), anti-αSMA followed by AlexaFluor 488 (visualized in green), and anti-mMCP6 antibody followed by AlexaFluor 594 secondary antibody (visualized in purple). Cell nuclei were counterstained with DAPI (blue). Images were acquired with a Zeiss LSM980 confocal microscope using a 20X/0.8NA objective or a 63X/1.3NA objective (inserts). **E** BMMCs were stimulated for 24 h with plastic-bound anti-CD44 in the presence of Brefeldin. The expression of IL-6, TNF-α, and VEGF was evaluated by flow cytometry upon permeabilization and intracellular staining. Representative histograms of unstimulated (red) and CD44-stimulated (orange) cells are shown in the upper panels together with isotype-matched Ctrl-Ig (blue). Means ± SD from three independent experiments are reported in the lower panels. Paired Student’s *t* test: *P < 0.05.

To investigate how MCs functionally adapt during CRC progression, we integrated our tumor dataset with a previously published AOM/DSS single-cell dataset, which includes additional cell populations, such as fibroblasts, endothelial cells, and diverse epithelial subsets [26]. The integrated UMAP representation revealed clear segregation of major cell populations (Fig. 4A) identified by their specific markers (Fig. S5A). We analyzed cell–cell communication and demonstrated that TAMCs have a high number/strength of interactions with cancer-associated fibroblasts (CAFs) (Fig. 4B, C), whose identity was confirmed based on gene expression (Fig. S5B). This close association was further supported by confocal microscopy analysis, which revealed direct contacts between TAMCs and fibroblasts (Fig. 4D). Communication between MCs and CAFs involved growth factors, immunoregulatory mediators, and numerous ECM-related signaling pathways (Fig. 4C and Table S2). Notably, interaction analysis identified CD44 as a major putative hub within this crosstalk, suggesting that CD44-mediated signaling may play a key role in orchestrating MC-CAF communication in the TME. Consistent with this hypothesis, CD44 engagement in vitro promoted the production of the pro-inflammatory cytokines IL-6 and TNF-α, as well as of the pro-angiogenic factor VEGF, in bone marrow-derived MCs (BMMCs) (Fig. 4E).

Of note, MCs also engaged in multifaceted interactions with endothelial and epithelial cells (Fig. S5C and Table S2).

Together, these findings indicate a bidirectional crosstalk between TAMCs and stromal cells within the TME, whereby stromal cues shape MC functions and TAMCs contribute to tissue remodeling and tumor progression.

### Tumor-associated MCs undergo functional reprogramming

**Fig. 5:**
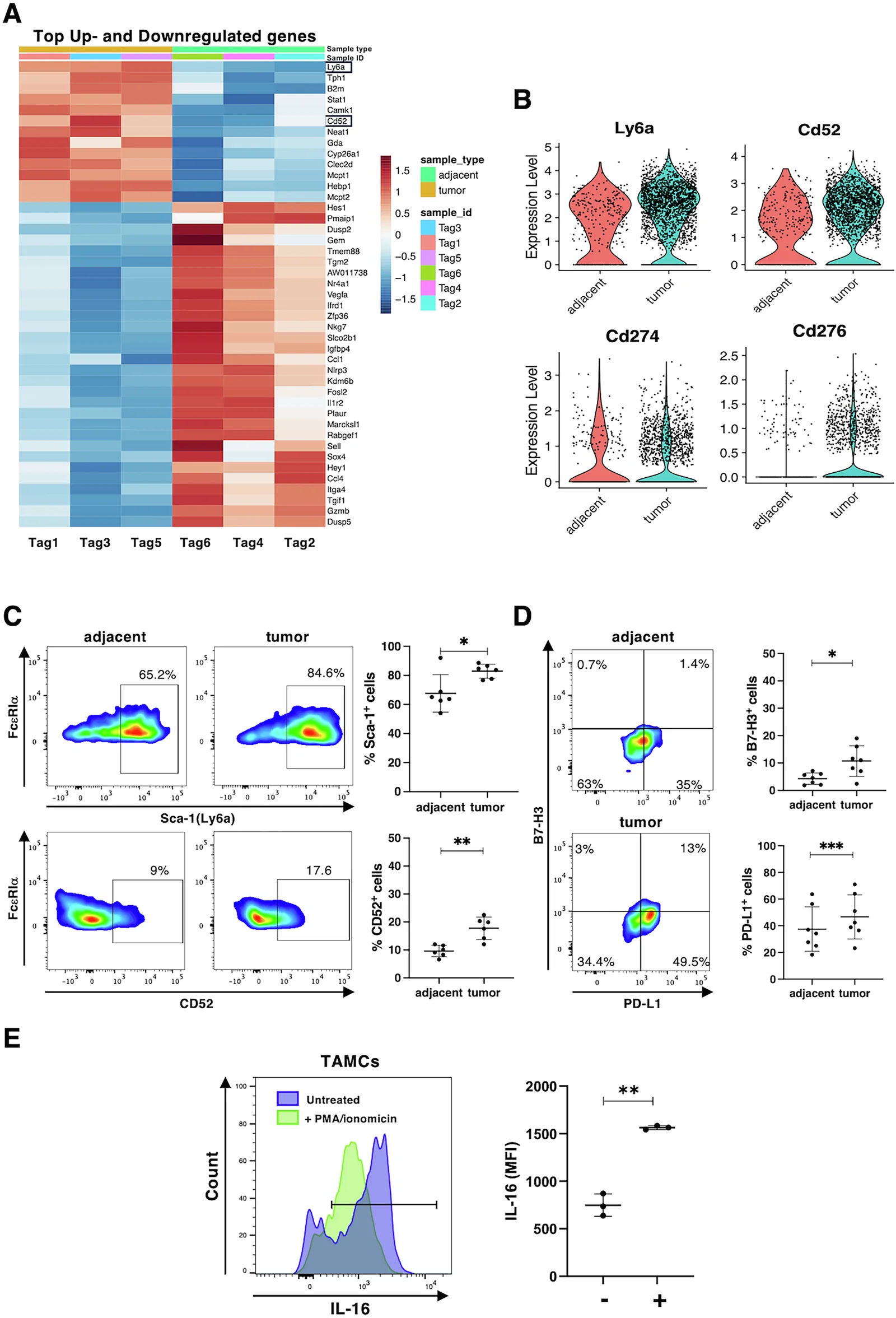
Upregulated genes and enriched biological pathways in MCs from tumor and adjacent non-transformed tissue. **A** Heatmap representation of upregulated and downregulated genes in MCs from tumor and adjacent non-transformed tissue obtained by pseudobulk analysis. Three replicates are characterized by a pool of 6 mice per group. Each group is called Tag 1, 3, 5 to refer to TAMCs, while Tag 2, 4, 6 refers to adjacent non-transformed tissue MCs. **B** Violin plot representation of Ly6a, Cd52, Cd274, and Cd276 transcripts in adjacent non-transformed and tumor tissue. **C**, **D** Flow cytometry analysis of Sca-1(Ly6a), CD52, PD-L1 (Cd274) and B7-H3 (Cd276) expression on MCs isolated from adjacent non-transformed and tumor tissue. Representative plots on the left and statistical analysis on the right of each panel. The expression level of surface markers was observed on six replicates (Sca-1 and CD52) and on seven replicates (PD-L1 and B7-H3). Paired Student’s *t* test: ***P < 0.001, **P < 0.01, and *P < 0.05. **E** Cells isolated from tumor tissues were left untreated (−) or stimulated with PMA/Ionomycin (+) for 2 h. MCs were identified based on c-kit^+^ FcεRIα^+^ markers, and the expression of IL-16 was analyzed upon permeabilization by flow cytometry. A representative experiment is shown on the left. The mean fluorescence intensity of IL-16^+^ MCs from three experiments with two mice/group is shown on the right. Paired Student’s *t* test **P < 0.01.

To define the molecular features of TAMCs, we performed pseudobulk differential expression analysis comparing MCs isolated from tumor- and adjacent non-transformed tissue across three biological replicates. TAMCs displayed extensive transcriptional reprogramming, characterized by the downregulation of multiple genes, including transcription factors, and concurrent upregulation of surface markers linked to immune modulation and tissue remodeling (Fig. 5A). Among these, *Ly6a* and *Cd52* were consistently elevated in TAMCs across all replicates, as highlighted by violin plots (Fig. 5B, upper panel). In addition, transcripts encoding the immune checkpoint PD-L1 (*Cd274)* and B7-H3 (*Cd276*) were modestly upregulated in TAMCs (Fig. 5B, lower panel). In line with these transcriptional changes, flow cytometry confirmed increased surface expression of Sca-1 (Ly6a), CD52 (Fig. 5C), PD-L1, and B7-H3 (Fig. 5D) in TAMCs, indicating acquisition of a distinct immunoregulatory phenotype.

To determine whether tumor-derived signals are sufficient to induce this TAMC phenotype, we co-cultured BMMCs with the CRC cell line MC38 for 48 hours. Tumor-exposed BMMCs acquired an activated phenotype, as evidenced by increased expression of the degranulation marker CD107a and the activation marker CD69 (Fig. S6A). Moreover, co-culture induced a decrease in α4 integrin and an increase in αE integrin expression (Fig. S6B), recapitulating the integrin profile observed in TAMCs in vivo (Fig. 3). Importantly, MC38 cells also promoted the upregulation of Sca-1 and PD-L1 (Fig. S6C), mirroring key phenotypic features of TAMCs.

Together, these findings indicate that tumor-derived cues are sufficient to drive key aspects of TAMC reprogramming, including both activation-associated and immunoregulatory traits.

To assess the functional consequences of this reprogramming, we analyzed pro-inflammatory cytokine expression across MC populations. While *Tnf* transcripts were enriched in MCs derived from adjacent non-transformed tissue, *Il6* expression was higher in TAMCs (Fig. S7A), consistent with a shift toward a pro-tumorigenic inflammatory phenotype previously described in the TME [21]. Notably, *Il16* transcripts were selectively elevated in TAMCs, with predominant expression in cluster 0 (Fig. S7B). Protein-level validation by intracellular staining confirmed constitutively high IL-16 expression in TAMCs, which further increased upon PMA/ionomycin stimulation (Fig. 5E), identifying IL-16 as a key TAMC effector potentially involved in immune cell recruitment within the TME.

**Fig. 6:**
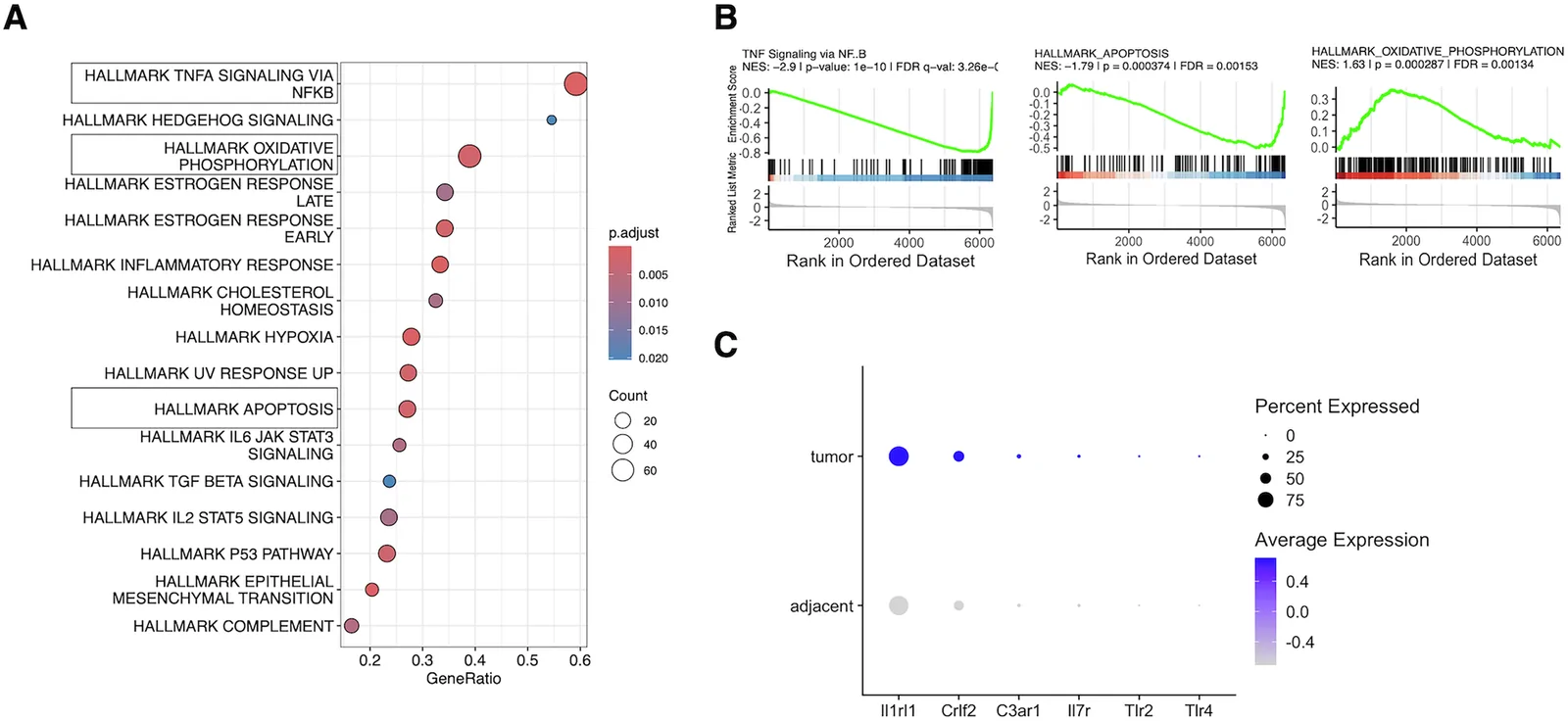
Pathway enrichment and surface marker expression in TAMCs. **A**, **B** Gene Set Enrichment Analysis (GSEA) showing significantly enriched hallmark pathways in MCs from tumor versus adjacent non-transformed tissue. **A** Dot plot represents the enriched pathways identified by GSEA. The size of the dot corresponds to the number of overlapping genes within each pathway, and its color indicates statistical significance (adjusted *P* value), as indicated in the legend. **B** The normalized enrichment score (NES) and False Discovery Rate (FDR) of the pathways highlighted in A are shown. Blue and red color gradients represent downregulation and upregulation in the tumor versus the adjacent tumor-free tissue. **C** Dot plot represents the expression of selected surface markers in MCs. Brightness indicates log-normalized average expression, and circle size indicates the percent of cells expressing each marker, as indicated in the legend.

To integrate these phenotypic and functional changes, we performed pathway enrichment analysis of differentially expressed genes from tumor and adjacent non-transformed samples (Fig. 6A). This analysis revealed an attenuation of TNF signaling via NF-κB (74 genes) and apoptosis-related genes (26 genes) (Fig. 6B), suggesting a transcriptional program that may favor TAMC survival and persistence within the tumor niche. Conversely, TAMCs exhibited a significant upregulation of the oxidative phosphorylation (OXPHOS) pathway (67 genes), indicating a metabolic shift toward mitochondrial respiration. Tumor-associated MCs showed increased expression of selected inflammatory and alarmin-associated receptors, including Il1rl1, Cx3cr1, Crlf2, C3ar1, Tlr2, and Tlr4, whereas Il7r was largely comparable, and Il10rb and Il6ra were reduced compared with adjacent MCs. This adaptation aligns with the observed increased expression of the IL-33 receptor (Il1rl1) and the thymic stromal lymphopoietin (TSLP) receptor (Cfrl2) (Fig. 6C), known to promote MC survival and metabolic adaptation [27, 28]. Notably, both receptors were enriched within cluster 0 (Fig. S7C), further supporting the notion that it represents a metabolically adapted, tumor-conditioned TAMC subset.

**Fig. 7:**
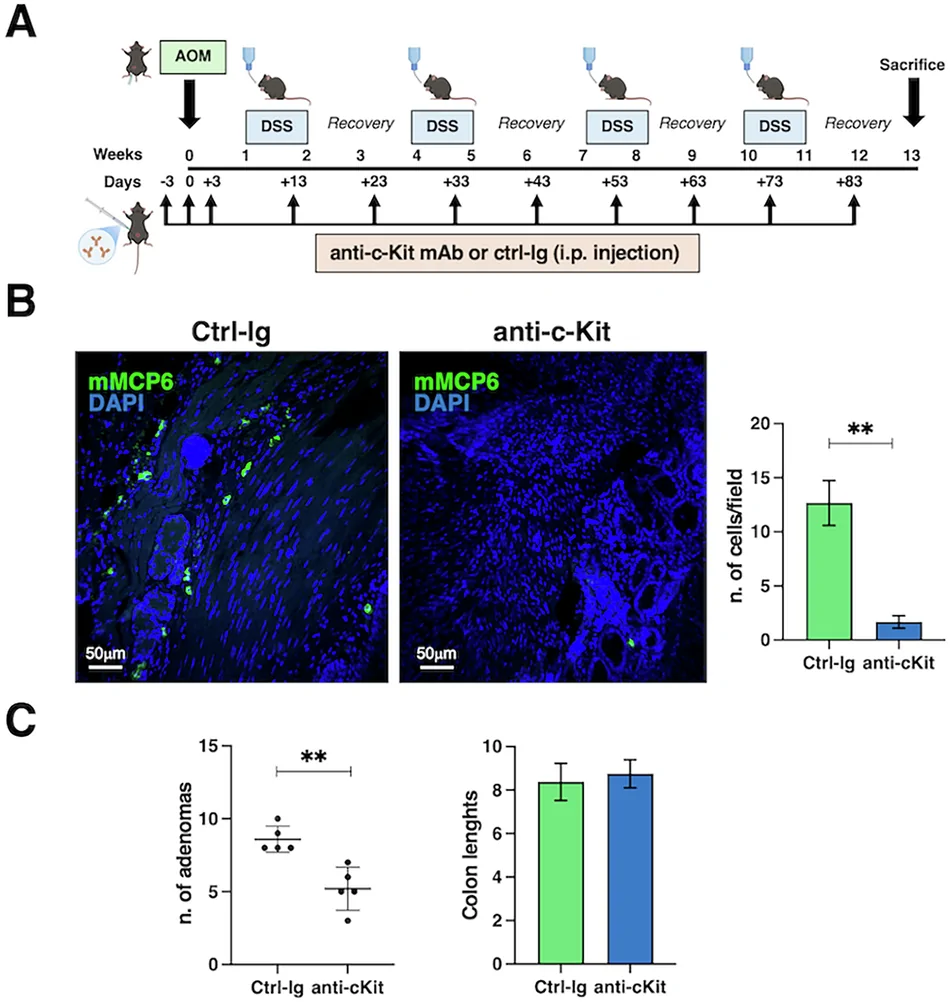
MC depletion reduces tumor burden. **A** Schematic representation of anti-cKit antibody administration in vivo. **B** Colon paraffin-embedded sections from AOM/DSS mice treated with Ctrl-Ig or anti-cKit neutralizing antibodies as described in A were stained with anti-MCP6 antibody followed by Alexa-fluor 488 secondary antibody (green). Nuclei were counterstained with DAPI (blue), and images were acquired with a Zeiss LSM980 confocal microscope using a ×20 objective. Fluorescence images randomly acquired from tumor lesions (20 fields from three independent experiments) were quantified and shown as means ± SD cells/field on the right. Data are representative of five mice/group. Paired Student’ *t* test: **P < 0.01. **C** The number of adenomas (left) and colon lengths (right) from five mice/group analyzed are shown. Paired Student’ *t* test: **P < 0.01.

To directly assess the role of MCs in CRC development, we depleted MCs during AOM/DSS-induced tumorigenesis using the anti-c-Kit monoclonal antibody ACK2 [29] (Fig. 7A). Efficient MC depletion, confirmed by the near-complete loss of mMCP6⁺ cells (Fig. 7B), significantly reduced tumor burden without affecting colon length (Fig. 7C).

Together, these results demonstrate that MCs promote CRC progression and support a model in which the TME reprograms MCs toward an immunosuppressive and pro-tumorigenic phenotype.

### Conserved tumor-associated MC states in human CRC

**Fig. 8:**
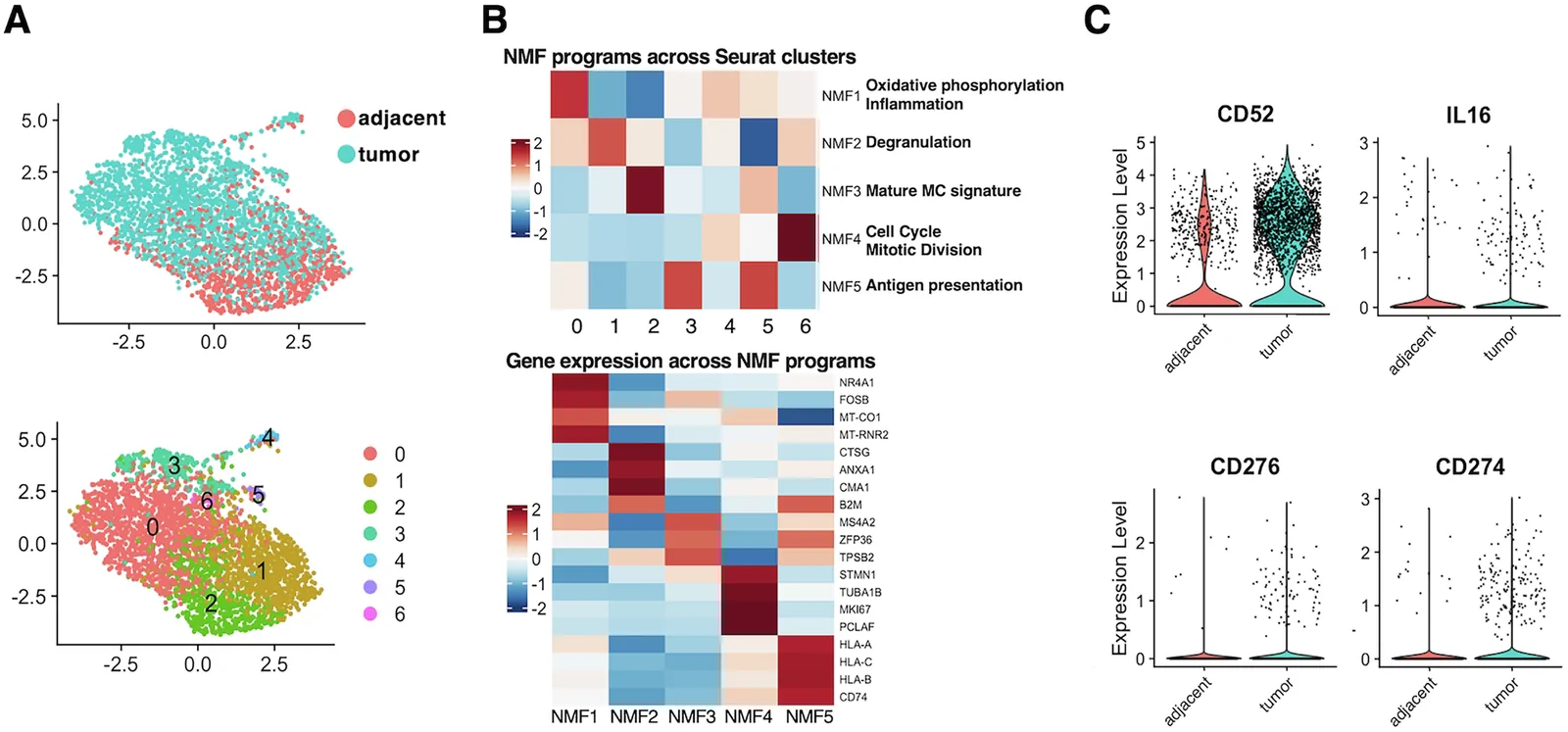
Human MC demonstrate comparable signature to murine MCs. **A** UMAP plot of human MCs colored by sample type (upper) and Seurat clusters (lower). **B** Heatmap of NMF1–NMF5 scores in MCs separated by Seurat clusters (upper) and of different genes across programs (lower). **C** Violin plots of CD52, CD274, CD276, and IL16 expression in MCs from tumor and tumor-free adjacent tissue.

To assess whether the tumor-associated MC states identified in the murine model are conserved in human disease, we analyzed single-cell RNA-seq data from the human CRC atlas [30]. MCs were subsetted based on canonical protease expression (*TPSAB1*, *TPSB2*, *CPA3*), and unsupervised clustering identified seven MC clusters (0-6). Cluster distribution differed between tumor and adjacent tissue (Fig. 8A), with tumor dominated by cluster 0 (54.6%) and cluster 3 (10%), while adjacent tissue was enriched in clusters 1 (41.9%) and 2 (37.3%). Clusters 4-6 were less abundant and similarly distributed between conditions (data not shown). To define the underlying transcriptional programs, we applied NMF, identifying five gene expression programs (Figs. 8B and S8 and Table S3). The OXPHOS and inflammation program (NMF1) was strongly associated with cluster 0 (Fig. 8B, upper panel), which predominates in the TME, mirroring the murine TAMC phenotype and indicating conserved metabolic reprogramming. Cluster 3, also enriched in tumors, was associated with an antigen presentation-related program (NMF5), highlighting an immunomodulatory role for a subset of TAMCs. In contrast, NMF2 and NMF3 were predominantly expressed in MCs from adjacent non-transformed colon tissue and were enriched for mature MC-associated genes, consistent with a homeostatic MC state.

Finally, analysis of surface and secreted factors revealed that human TAMCs exhibited elevated expression of *IL16, CD52, CD274* (PDL-1), and *CD276* (B7-H3) transcripts (Fig. 8C), supporting the existence of a conserved, tumor-conditioned TAMC state across species.

## Discussion

In this study, we provide a comprehensive single-cell characterization of MCs in a model of inflammation-associated CRC. By analyzing cells from both tumor and adjacent non-transformed tissue, we reveal significant changes in MC abundance, phenotype, and function within the TME. In agreement with our previous report [21], we demonstrate the highest frequency of MCs within tumor tissue. Importantly, transcriptomic analysis further reveals that TAMCs comprise diverse subpopulations with distinct transcriptional states. The most represented TAMC subset, cluster 0, is fully differentiated and characterized by elevated expression of mMCP6 protease (Figs. 1, 2).

Notably, within tumor tissues, we have previously observed an enrichment in IL-33, which represents a key factor driving the polarization of MCs toward a mMCP6^+^ phenotype [21]. Accordingly, an earlier study demonstrated a selective expansion of mMCP6⁺ MCs in small bowel cancer, firmly supporting a pro-tumoral role for this subset [31]. In line with this, recombinant mMCP6 has been shown to promote angiogenesis by acting on endothelial cells [32]. Together, the tumor enrichment of mMCP6-expressing MCs (Fig. 1F), the activation of proteolysis- and stimulus-response pathways (Fig. 1G), and the presence of MC-endothelial interaction signatures (Fig. S5C) suggest that MCs actively drive vascular remodeling in the TME.

Beyond vascular crosstalk, MCs can also activate fibroblasts into CAFs, which in turn promote extracellular matrix remodeling and further MC recruitment [33]. Consistently, TAMCs emerged as one of the cell populations most strongly associated with CAFs (Fig. 4B, C), a finding further supported by confocal microscopy (Fig. 4D) demonstrating their close physical interaction within the TME. Interestingly, the MC-CAF crosstalk appeared to be largely orchestrated by CD44-dependent signaling (Fig. 4C). Given that CD44 engagement induced the production of pro-inflammatory cytokines and promoted the pro-angiogenic functions of MCs (Fig. 4E), this pathway may represent a key mechanism through which CAFs shape TAMC activity within the TME.

Thus, reciprocal interactions between TAMCs and CAFs may orchestrate TME remodeling to sustain tumor progression.

We also highlighted different expression levels of integrins among MC populations (Fig.3). However, the specific contribution of individual integrins to MC localization and interactions with stromal cells and/or other immune cells remains to be determined. In particular, the possibility that integrin-dependent pathways modulate MC crosstalk with macrophages and T cells represents an important area for future investigation.

With respect to MC reprogramming, gene set enrichment analysis (GSEA) indicates that TAMCs undergo a metabolic shift toward OXPHOS, concomitant with repression of apoptosis (Fig. 6A, B). Notably, TAMCs express high levels of the IL33 receptor and the TSLP receptor (Fig. 6C). IL-33 has been shown to drive metabolic changes required for MC degranulation, including upregulation of the OXPHOS pathway [28], whereas TSLP acts as a potent pro-survival cytokine for MCs [27]. Collectively, these findings support a model in which IL-33- and TSLP-mediated signaling promotes metabolic reprogramming of MCs, sustaining their survival and functional adaptation within the TME.

GSEA further revealed downmodulation of TNF-α/NF-κB signaling in TAMCs (Fig. 6), consistent with reduced *Tnf* transcript levels in tumor-associated compared with adjacent tissue MCs (Fig. S7A). Although TNF-α can support tumor growth when present at low, chronic levels, elevated TNF-α preferentially activates TNFR1-dependent apoptotic and senescence pathways. Thus, reduced TNF-α expression within TME likely reflects selective pressure to evade TNF-α-mediated cytotoxicity, while preserving pro-tumorigenic inflammatory signaling [34–37].

TAMCs showed increased PD-L1 expression (Fig. 5B, D), consistent with an immunosuppressive phenotype in the absence of overt inflammation. This evidence, also confirmed in vitro (Fig. S6C), aligns with reports in human gastric cancer and contact hypersensitivity models, where PD-L1-expressing MCs suppress CD8⁺ T-cell responses [38, 39]. Notably, we identify B7-H3 expression by MCs in CRC for the first time, suggesting that B7-H3/PDL-1 co-expression by TAMCs may contribute to reinforcing local immunosuppression.

Consistent with this immunosuppressive phenotype, we observed marked upregulation of CD52 (Fig. 5), a molecule known to suppress T-cell effector functions and to mark polymorphonuclear myeloid-derived suppressor cells [40, 41]. Notably, CD52 expression was strongly enriched in cluster 0 (Fig. 1G), further implicating this subset in shaping an immunosuppressive TME.

Collectively, these findings support a pro-tumorigenic, immunosuppressive role of TAMCs in CRC, reinforcing prior evidence from MC-deficient mouse models [15, 42–45]. Accordingly, antibody-mediated MC depletion reduced tumor growth (Fig. 7C), further validating the pro-tumoral MC reprogramming in TME. These findings identify TAMCs as potential therapeutic targets and suggest that disrupting TAMC accumulation or function may represent a novel immunotherapeutic strategy for CRC.

Among TAMC-derived cytokines, we report for the first time IL-16 expression at both the transcript and protein levels (Figs. S7A and 5E). This finding is consistent with elevated IL-16 in MCs from inflamed Crohn’s disease mucosa, where it promotes CD4⁺ T-cell recruitment [46], as well as with evidence that IL-16 neutralization attenuates colonic inflammation in mice [47]. Together, these observations suggest that TAMCs act as a dual modulator of T-cell response, promoting recruitment via IL-16 while constraining effector function through checkpoint-mediated inhibition. This combined activity may foster the accumulation of dysfunctional or suppressed T cells within the TME, contributing to immune evasion in CRC. Importantly, the majority of these transcriptional and functional features are conserved in human CRC (Fig. 8), underscoring the biological relevance of MC reprogramming beyond experimental systems. Collectively, our findings reveal the remarkable plasticity of MCs in CRC and position them as key drivers of tumor progression, immune escape, and TME remodeling. Targeting MC-derived immunoregulatory pathways may therefore offer a previously unrecognized therapeutic option.

## Materials and methods

### Murine model

All animal procedures were approved by the Italian code for the care and use of animals for scientific purposes (Authorization no. 698/2021-PR).

Six-week-old female C57BL/6J mice were purchased from Charles River (Wilmington, MA). Colonic tumors were induced using AOM/DSS treatment, as previously described [21].

For MC depletion, mice were intraperitoneally injected with 200 μg of ACK2 (Cat# 135132; BioLegend, San Diego, CA) or a Rat IgG2b, κ Isotype Ctrl Antibody (Cat# 400644; BioLegend) in 200 μl of sterile PBS three days before, together with AOM administration and three days after AOM. Injection was then repeated every ten days for the duration of DSS treatment. Treated and control mice were sacrificed at 13 weeks after AOM administration.

### Cell isolation and cell sorting

Colons were dissected, cut longitudinally, and tumors were separated from the adjacent tissue. We obtained three replicates for each sample, including 6 mice per group, for a total of 18 mice. Samples were processed as previously reported [21]. Leukocytes were enriched using 40% Percoll (Merck Life Sciences, Milan, Italy) followed by another enrichment step using a cocktail of biotinylated antibodies against NK1.1 (Cat# 553163, BD Biosciences, San Jose, CA, USA), CD3 (Cat# 130-131-559, Miltenyi Biotec, Bergisch Gladbach, Germany), CD19 (Cat# 130-126-041, Miltenyi Biotec) and CD11b (Cat# 13-0112-85, Thermo Scientific, Waltham, Massachusetts, USA). Labeled cells were removed with streptavidin-coated microbeads (Thermo Scientific). The remaining cells were stained with APC-H7-conjugated Fixable viability Dye (Thermo Scientific), and FcεRIα/c-Kit double and single positive cells were sorted after excluding TCRβ^+^/B220^+^/NK1.1^+^/Cd11b^+^/dead cell and doublets using the gating strategy shown in Fig. S1. Antibodies used are listed in Table S4.

To reduce stress, cells were isolated in gentle conditions using a ceramic nozzle of size 100 mm, a low sheath pressure of 19.84 pound-force per square inch (psi) that maintained the sample pressure at 18.96 psi using a FACSAriaIII (BD Biosciences) equipped with Near UV 375, 488 nm, 561 nm, and 633 nm lasers and FACSDiva software (BD Biosciences version 6.1.3). Sorted cells were confirmed to be highly purified in the post-sorting analysis.

### Single-cell RNA sequencing library preparation

Sorted c-kit and FcεRIα single and double-positive cells were labeled using Single Cell Labeling with the BD Single-Cell Multiplexing Kit (Cat# 633793, BD Biosciences). Briefly, cells were labeled with sample tags, and each sample was washed twice with Stain buffer. Samples were pooled, resuspended in cold BD Sample Buffer, and counted in a hemocytometer, staining the cells with Calcein AM and Draq7TM. Cells were then isolated using Single Cell Capture and cDNA Synthesis with the BD Rhapsody Express Single-Cell Analysis System following the manufacturer's protocol. cDNA Libraries were prepared using mRNA whole transcriptome analysis (WTA) and the Sample Tag library preparation protocol. Final libraries were diluted to 4 nM and multiplexed for paired-end (2 × 75 bp) sequencing on a NovaSeq +q6000TM sequencer (Illumina, San Diego, CA, USA). Sequence reads were aligned to the reference mouse genome mm10 (UCSC), following generation of barcode-gene matrices via the pipeline “BD Rhapsody™ WTA Analysis” from SevenBridges (https://www.sevenbridges.com/).

### Single-cell RNA seq data processing

Reads were trimmed using cutadapt v4 and were mapped using STAR (v2.7.1) [48] to the Mus_musculus GRCm38, and index files were produced with Samtools. The resultant dataset was converted into a Seurat object using R Seurat package (version 5.1.0) [49]. Primary clusters were identified using the “FindNeighbors” and “FindClusters” functions, with a resolution of 0.2. During the first round of clustering, cell types are identified and labeled based on the expression of known marker genes as reported in Fig. S2A. For MC lineage, the cells were subjected to re-clustering using a resolution of 0.5 employing 20 principal components. We utilized the “Findallmarkers” function in Seurat and considered as differentially expressed genes (DEGs) those that met the following criteria: log fold-change threshold=0.25, min.pct=0.2.

We employed an NMF analysis [50] for the identification of transcriptional programs and selected a rank of 5, as beyond this value, we did not observe any differences between programs. We extracted the top 25 genes with the highest weights (Table S1) by performing the NMF algorithm: “brunet” and random seeding method, calculating 2000 interactions. Using clusterProfiler package (v4.10.1) [51], we identified the pathways activated in each program.

### Pseudotime trajectory analysis

To investigate the developmental trajectory of different MC subsets, we employed the “monocle3” package (v1.3.5) [52] according to the authors’ guidelines (https://cole-trapnell-lab.github.io/monocle3/). CytoTRACE2 analysis was performed using the CytoTRACE2 package (v1.1.0) in R [53]. The normalized gene expression matrix obtained from the Seurat object was used as input, and developmental potency scores were calculated according to the authors’ recommendations. CytoTRACE2 assigns each cell a score reflecting its predicted differentiation state, with higher scores indicating greater developmental potential and a less differentiated phenotype.

### Cell–cell communication analysis

To explore potential interactions between MC and other cell types within the TME, we first integrated the single-cell RNA-sequencing datasets using the Harmony package (v1.2.3). The resulting integrated object was used as input for CellChat (v2.2.0) [54], and the analysis was performed following the standard CellChat workflow. Only significant ligand-receptor pairs (*P* < 0.05) were considered for downstream interpretation.

### Pseudobulk differential expression and gene set enrichment analysis

To compare transcriptional programs between MCs from tumor and adjacent non-transformed tissue, we performed a pseudobulk differential expression analysis. MCs were grouped by sample origin (adjacent non-transformed or tumor tissue), and for each sample, gene expression counts were aggregated (summed) to create a pseudobulk profile. Differential expression analysis between adjacent non-transformed and tumor-derived MCs was performed using DESeq2 (v1.42.1) with default parameters (http://bioconductor.org/packages/devel/bioc/vignettes/DESeq2/inst/doc/DESeq2.html). Genes with an adjusted *P* value < 0.05 and absolute log2 fold change >0.5 were considered significantly differentially expressed. To identify pathways specifically upregulated in TAMCs, we conducted Gene Set Enrichment Analysis GSEA [55] using the clusterProfiler package (v4.10.1) [56] in R. Enrichment was tested against the KEGG gene sets. Pathways with a false discovery rate (FDR) < 0.05 were considered significantly enriched. For selected pathways of interest, we performed single-curve enrichment analysis to visualize the running enrichment score of each gene set along the ranked gene list. This allowed us to assess the distribution and relative contribution of individual genes within specific pathways to the overall enrichment signal. Enrichment plots were generated using the gseaplot2 function from the enrichplot package (v1.22.0) [55, 57].

### Human single-cell analysis

For the human MC dataset, we utilized the colon cancer atlas generated by Pelka and coauthors [30] and subsetted MCs based on the expression of TPSB2, TPSAB1, and CPA3.

MCs were used to create a Seurat object and processed following the same workflow described for the mouse dataset. To identify conserved transcriptional programs, we applied NMF to the human MC expression matrix using the same parameters as in the mouse analysis (rank = 5). For each resulting program, we extracted genes with the highest NMF weights (Table S3) and performed pathway enrichment analysis.

### Immunofluorescence analysis

Colons were Swiss-rolled, fixed, and embedded in paraffin, as previously described [21]. Staining was performed as previously described [21] using the following primary and secondary antibodies: goat anti-mMCP4 (Cat# ab92368, Abcam, Cambridge, UK) and AlexaFluor 488-conjugated donkey anti-goat antibody; goat/rat anti-mMCP6 (Cat# AF3736/MAB3736, R&D Systems, Minneapolis, MN, USA) and AlexaFluor 594-conjugated donkey anti-goat/-rat antibody; goat anti-mouse α-SMA (Cat# Ab7817, Abcam) and AlexaFluor 488-conjugated donkey anti-goat antibody; rabbit anti-integrin αE antibody (Cat# ab224202, Abcam) and AlexaFluor 488-conjugated goat anti-rabbit antibody; rat anti-CD31 (Cat# DIA310, Dianova) and Alexa Flour 647-conjugated donkey anti-rat antibody. An intermediate step of permeabilization with 0.5% Triton for 20min at room temperature, after antigen retrieval, was performed before integrin staining. Nuclei were counterstained by DAPI. Images were acquired using a Zeiss LSM980 confocal microscope equipped with ×20/0.8NA and 63X/1.35NA objectives. Images were processed using Fiji software.

### Flow cytometry analysis

Cells isolated from tumor masses and adjacent tumor-free tissues were stained for surface markers using the antibodies listed in Table S4. Dead cells were excluded using an APC-H7–conjugated Fixable Viability Dye (Thermo Scientific). For IL-16 detection, leukocytes isolated from tumor masses were either stimulated with PMA/ionomycin (Cat# 00497093, Thermo Scientific) or left unstimulated in the presence of Brefeldin A (Cat # B7651, Merck) for 2 h. After stimulation, cells were fixed and permeabilized using the BD Cytofix/Cytoperm™ Fixation/Permeabilization Kit (Cat# 554714, BD Biosciences) for 25 min, then blocked with goat serum for 15 min at 4 °C to prevent nonspecific binding. Intracellular staining for IL-16 was performed using a rabbit anti-IL-16 antibody (Cat# PA5-79469, Thermo Scientific) followed by an AlexaFluor 647-conjugated goat anti-rabbit secondary antibody (Thermo Scientific). Samples were acquired by the LSR Fortessa Flow cytometer, and the analysis was performed with FlowJo 10 software (BD Bioscience).

### Co-culture and in vitro stimulation

Bone marrow cells were isolated from the femurs of C57BL/6 mice and differentiated as previously described [58]. The murine colon adenocarcinoma MC38 cell line was maintained in RPMI-1640 supplemented with 10% FCS. For co-culture experiments, BMMCs were incubated with MC38 tumor cells at a 1:2 ratio (BMMCs:MC38) for 48 h. Brefeldin A was added for the last 3 h. BMMCs cultured alone were used as a negative control. Following co-culture, BMMCs were harvested, stained for Fixable Viability Dye, FcεRI, and CD117 to gate live MCs and the expression of integrin α4 (CD49d), αE (CD103), CD107a, CD69, Sca-1, and PD-L1 using the antibodies listed in Table S4. CD44 stimulation of BMMCs was performed by adsorbing 5 μg/ml of purified anti-mouse/human CD44 (BioLegend; Cat# 103002; Clone: IM7) to 24-well plates at 4 °C overnight. Plates were then washed twice in PBS. BMMCs were subsequently seeded (2.5 × 10^5^ cells/ml) and incubated for 24 h. Brefeldin A was added for the last 3 h. BMMCs cultured in non-coated wells were used as a negative control. After stimulation, cells were collected, and the expression of IL-6, TNF-α, and VEGF was analyzed by flow cytometry upon fixation and permeabilization. Antibodies used are listed in Table S4.

### Statistical analyses

Statistical analyses were conducted using Prism (GraphPad Software). Student’s *t* test was used for comparison between two groups. Graphs show mean values, and all error bars represent the SD. Asterisks denote significant differences. For the analysis of pseudobulk sequencing data, FDR correction for multiple comparisons was used.

## Supplementary information

Supplementary data

## Data Availability

Human mast cells were obtained from GSE178341. The AOM/DSS data integrated were obtained from Gene Expression Omnibus (GEO) at GSE134255; the samples included in the analysis correspond to GSM3940486, GSM3940487, and GSM3940488. The row and processed data generated in this study have been deposited in the GEO under accession number GSE313794.

## Code availability

All scripts used for preprocessing, single-cell analysis, pseudobulk analysis, cell-to-cell communication, NMF program identification, and downstream visualization are publicly available at: https://github.com/erisap/MCs-and-CRC.
