# Supplementary material for: Single-cell RNA sequencing reveals pro-tumorigenic intestinal mast cell programs in colorectal cancer
Source: Cell Death Dis. 2026 Aug 12;17(1):968. doi: 10.1038/s41419-026-09183-1 (PMC13768332; doi:10.1038/s41419-026-09183-1)
Supplement: Supplementary file 1 [file 41419_2026_9183_MOESM1_ESM.docx]

**Supplementary Figures**

**Figure S1. Flow cytometric gating strategy for sorting of FcεRIα and/or c-Kit positive cells.**

*Cells isolated from tumor and adjacent tumor-free colon tissues were first gated based on morphologic characteristics. We first excluded dead cells (APC-H7-conjugated Fixable viability Dye) and TCRβ^+^/B220^+^/NK1.1^+^/Cd11b^+^ (all FITC-conjugated Abs), followed by exclusion of doublets with morphology parameter area versus width (A versus W). Finally, leukocytes positive for FcεRIα and/or c-Kit were sorted.*

**Figure S2. Signature genes and cell populations in AOM/DSS treated mice.**

***A*** *Dot plot graph showing the expression of selected marker genes across identified cell types, including one cluster of MCs (Cpa3), three clusters of ILCs (Il7r), one cluster of basophils (Mcpt8), two clusters of DCs (Flt3), one cluster of epithelial cells (Chdr2), one cluster of Neutrophils (S100a9), one cluster of pre-B lymphocytes (Igkv8-24) and one cluster of neuroendocrine cells (Chga). Brightness indicates log-normalized average expression, and circle size indicates the percentage expressed as indicated in the legend to the right.* ***B*** *Violin plot depicting the expression of MC-specific proteases Mcpt1, Cpa3 and Tpsb2 among all cell types of the dataset. Each color of the violin plot represents a distinct cell population, as indicated in the legend.*

**Figure S3. Characterization of mature TAMCs and transcriptional profile of MCp.**

***A*** *Colon paraffin-embedded sections from AOM/DSS treated mice were stained with anti-mMCP6 and anti-mMCP4 followed by Alexa Fluor 594 (red) and 488 (green) -conjugated secondary antibodies, respectively. Nuclei were counterstained with DAPI (blue). Images were acquired with a Zeiss LSM980 confocal microscopy using a 63X/1.3NA objective. Yellow spots represent colocalization.* *Quantification was performed in seven randomly selected tumor fields, and the number of mMCP4⁺ single-positive and mMCP4⁺mMCP6⁺ double-positive MCs was quantified with Fiji ImageJ and analyzed using GraphPad Prism.* ***B*** *Dot plots showing specific marker gene expression levels related to basophil and MC precursors (Mcpt8, Cd9, Stat5b) populations and mature basophil markers (Cebpa, Itga2/Cd49b). Brightness indicates log-normalized average expression, and circle size indicates the percent expressed.*

**Figure S4. Transcriptional program distribution in tumor versus adjacent tumor free tissue MCs.**

*Violin plots showing the distribution of NMF scores across MCs isolated from adjacent (red) and tumor (blue) tissue. Each plot (NMF1-NMF5) represents one transcriptional program derived from NMF analysis, as indicated.*

**Figure S5. Single-cell transcriptomic identification of different cell populations obtained from integrated dataset and cell-cell interaction.**

***A*** *Dot plot graph demonstrates expression of different selected marker genes per cell type in the integrated dataset. Brightness indicates log-normalized average expression, and circle size indicates the percentage expressed as indicated in the legend to the right.* ***B*** *Violin plot representation of CAF population specific markers, among all cell types of the tumor integrated dataset.* ***C*** *CellChat cell-cell communication networks inferred from expression of ligand-receptor pairs. Chord diagrams showing communication between), MCs and Epithelial cells (left plot) and between MC and endothelial cells (right plot).*

**Figure S6. Surface marker expression on bone marrow-derived MCs upon co-culture with tumor cells.** *BMMCs were co-cultured with MC38 colorectal cancer cell line for 48 h at the 1:2 ratio. Cells were harvested and the expression of the activation markers CD107a and CD69 (****A****), integrin subunits α4 and αE (****B****), Sca-1 and PD-L1 (****C****) were evaluated by flow cytometry. Representative histograms of unstimulated (red) and MC38-stimulated (orange) cells are shown in the left panels together with isotype-matched Ctrl-Ig (blue). Means ± SD of three independent experiments are shown in the right panels.* *Paired Student’ t test: Paired Student’ t test: **p<0.01, *p<0.05.*

**Figure S7. Expression levels of cytokine and surface marker transcripts in TAMCs.**

***A*** *Violin plot representation of Tnf, Il6 and Il16 transcripts based on sample type (adjacent versus tumor). Violin plot of Il16 (****B****) and IL1rl1, Crlf2 (****C****) transcripts across MC clusters. Each color of the violin plot represents a specific cluster in the dataset.*

**Figure S8. Transcriptional program distribution in human MCs from tumor versus adjacent tumor free tissue.**

*Violin plots showing the distribution of NMF scores across MCs from adjacent (red) and tumor (blue) tissue in human colon cancer atlas. Each plot represents one transcriptional program derived from NMF analysis, as indicated.*

**Table S1: Highest expressed marker genes for each NMF transcriptional program identified in murine MCs**

These genes represent the most strongly enriched contributors to each program and correspond to the functional annotations shown in Fig. 1G.

| **NMF1** | **NMF2** | **NMF3** | **MNF4** | **NMF5** |
| --- | --- | --- | --- | --- |
| Egr1 | Ifitm1 | Tpsb2 | Stmn1 | Scin |
| Jumb | Actb | Cma1 | Hmgb2 | Actb |
| Nr4a1 | Igkc | Tpsab1 | Tuba1b | Ifitm1 |
| Tnfaip3 | Rgs1 | Actb | Top2a | Cfl1 |
| H3f3b | Actg1 | Mcpt4 | Ptma | Prma |
| Nfkbiz | H3f3b | Tpsg1 | Tubb5 | Rps26 |
| Fos | Ptma | Tph1 | H2az1 | Hsp90ab1 |
| Nfkbia | Fgl2 | Actg1 | Mki67 | Gzmb |
| Igkc | Itgae | Igkc | Smc4 | Rps2 |
| Dusp1 | Mt-Rnr1 | Rps26 | Tmpo | Hspa5 |
| Rgs1 | Rps26 | Serpinb1a | Plcaf | Actg1 |
| Fosb | Rgs2 | CD52 | Dek | Eif5a |
| Zfp36 | Efr1 | Cfl1 | Birc5 | Rpl31 |
| Ier2 | Hsp90ab1 | Ly6a | Nunks1 | Rps17 |
| Mcpt4 | Cfl1 | Rgs1 | Ccna2 | Hsp90aa1 |
| Actb | Ly6a | Ptma | Smc2 | Rps20 |
| Actg1 | Rps20 | Cd24a | Actb | Calr |
| Mt-Rnr1 | Jumb | H3f3b | Cenpe | Ybx1 |
| Ier3 | Btg1 | Rsp20 | Igkc | Serbp1 |
| Ccl2 | Plac8 | Nph22 | Anp32b | Hsp90b1 |
| Pim1 | Fos | Mt-Rnr1 | Pcna | Scl25a5 |
| Ier5 | Gm2a | Hsp90ab1 | Cks2 | Ran |
| Ptma | Ier5 | Vim | Ppia | Calm1 |
| Btg1 | Sat1 | Ifitm1 | Hmgn2 | Igkc |
| Tpsb2 | Cma2 | Btg1 | Csk1b | Ywhae |

**Table S2: Predicted ligand-receptor interactions of TAMCs with stromal and epithelial compartments**

This table summarizes the most relevant ligand-receptor pairs identified by CellChat analysis. Interactions are grouped by their functional category, including extracellular matrix (ECM) remodeling, growth signals, immunoregulatory pathways.

| **Functional category** | **MC-Endothelial cells** | **MC-Epithelial cells** | **MC-CAF** |
| --- | --- | --- | --- |
| **Adhesion/ECM remodeling** | Cd44-Col (collagen),  Cd44-Lam (laminin),  Cd47-Thsb | Itgae-Cdh1(E-cadherin), Cd44-Fn1, Cd74-App, Cd44-Lam/Lgals9 | Cd44-Collagen,  Cd44-Fn1, Cd44-Lam  Itga7-Lam, |
| **Survival/growth signals** | Kit-Kitl,  Tmem219-Igfbp3 | _ | Fgfg1-Fgf7,  Tgfbr1/r2-Tgfb2 |
| **Immunoregulatory pathways** | Cd200r3-Cd200, Cd44-Lgal (galectins) | Ptprc-Lgals9, Cd74_Cd44-Mif | Adgre5-Thy1,  Adgrg1-Mif |
| **Other mediators/signaling** | Sorl1*-*App, Cd74_Cd44-Mif | Thbs-Cd47 | Cd74-App |

**Table S3: Highest expressed marker genes for each NMF transcriptional program identified in human MCs.**

These genes represent the most strongly enriched contributors to each program and correspond to the functional annotations shown in Fig. 8B.

| **NMF1** | **NMF2** | **NMF3** | **MNF4** | **NMF5** |
| --- | --- | --- | --- | --- |
| MT-CO1 | TPSB2 | TPSB2 | STMN1 | HLA-B |
| MT-CO3 | B2M | TPSAB1 | H2AFZ | HLA-A |
| MT-CO2 | TPSAB1 | MT-CO1 | TUBA1B | HLA-C |
| EEF1A1 | MT-CO1 | MT-ND4 | TUBB | TPSB2 |
| TMSB4X | TMSB4X | B2M | PTMA | B2M |
| MT-RNR2 | MT-CO2 | TMSB4X | HINT1 | ACTB |
| MT-ND4 | MT-ND4 | MT-CO2 | HMGB1 | LGALS1 |
| B2M | MT-CO3 | MT-CO3 | HMGN2 | VIM |
| MT-ND3 | MT-ATP6 | EEF1A1 | HMGB2 | TPSAB1 |
| MT-ATP6 | SRGN | MT-ND3 | LGALS1 | CLU |
| TPSAB1 | EEF1A1 | MT-ATP6 | EXD3 | GADPH |
| TPSB2 | MT-CYTB | SRGN | GADPH | MT2A |
| NFKBIA | ACTB | MT-CYB | ACTB | EEF1A1 |
| BIRC3 | MT-ND3 | TPT1 | H2AFV | S100A11 |
| SRGN | ANXA1 | IGKC | DUT | CPA3 |
| TPT1 | S100A4 | CPA3 | MT2A | ACTG1 |
| CPA3 | NFKBIA | MT-RNR2 | PTTG1 | TMSB4X |
| TNFAIP3 | CPA3 | TMSB10 | PPIA | CD52 |
| BTG2 | TSMB10 | IGHA1 | PFN1 | TIMP1 |
| MT-CYB | MT-RNR2 | RPL7A | PZT2B | PFN1 |
| MT-ND1 | TPT1 | S100A4 | RAN | CFL1 |
| FOSB | UBB | ACTB | PCLAF | NSMCE1 |
| MT-RNR1 | CALM2 | MT-ND1 | CFL1 | RAC2 |
| RPL7A | CAPG | MS4A2 | HNRNPA2B1 | S100A4 |
| TMSB10 | MT-ND1 | VIM | HIST1H4C | CAPG |

**Table S4: Antibodies used in flow cytometry and cell sorting.**

| **Antibody** | **Cat #** | **Supplier** |
| --- | --- | --- |
| anti-FcεRIα APC | 134316 | BioLegend |
| anti-FcεRI-PE-Cy7 | 134318 | BioLegend |
| anti-FcεRI-Pacific Blue | 134314 | BioLegend |
| anti-c-Kit-PE | 105807 | BioLegend |
| anti-c-Kit-BV421 | 105827 | BioLegend |
| anti-c-Kit-FITC | 553354 | BD Biosciences |
| anti-CD45.2-PerCP-Cy5.5 | 45-0454-82 | Thermo Fisher Scientific |
| anti-NK1.1-FITC | 108706 | BioLegend |
| anti-CD11b-FITC | 11-0112-82 | Thermo Scientific |
| anti-CD19-FITC | 152404 | BioLegend |
| anti-CD3-FITC | 100306 | BioLegend |
| anti-TCRβ FITC | 109205 | BioLegend |
| anti-CD45R/B220-FITC | 103205 | BioLegend |
| anti-CD274-APC | 564715 | BD Biosciences |
| anti-CD276-BV510 | 740201 | BD Biosciences |
| anti-Sca-1-PE-CF | 562730 | BD Biosciences |
| anti-CD52-PE | 165705 | BioLegend |
| anti-Integrin-αE-BUV737 | 569678 | BD Biosciences |
| anti-Integrin-α4-BV786 | 564397 | BD Biosciences |
| anti-Integrin-α7-BV605 | 755154 | BD Biosciences |
| anti-CD107a-BUV395 | 565533 | BD Biosciences |
| anti-CD69-PE-Cy7 | 104512 | BioLegend |
| anti-IL-6-APC | 504507 | BioLegend |
| anti-TNF-α-BV510 | 506339 | BioLegend |
| anti-VEGFA-PE | 209439 | Abcam |
